# Supplementary material for: Nucleotide dependency analysis of genomic language models detects functional elements
Source: Nat Genet. 2025 Oct 10;57(10):2589–602. doi: 10.1038/s41588-025-02347-3 (PMC12513839; doi:10.1038/s41588-025-02347-3)
Supplement: Supplementary file 1 — Supplementary Note. [file 41588_2025_2347_MOESM1_ESM.pdf]

nature genetics

Supplementary information https://doi.org/10.1038/s41588-025-02347-3

Nucleotide dependency analysis of genomic language models detects functional elements

In the format provided by the authors and unedited

Supplementary Text

Analogous to antiparallel diagonals, we frequently observed parallel diagonals in the dependency maps, as seen in the promoter of *YNR064C* in *S. cerevisiae* (Fig. 5A), which exhibits a tandem repeat of two adjacent identical sequences of length 16. The parallel diagonal here reflects that the n-th nucleotide in one repeat predicts the n-th nucleotide in the other. This contrasts with the block pattern of regulatory motifs (e.g., TATA box, Fig. 5A), where all nucleotides are interdependent.

To study general properties of parallel diagonals, we first systematically scored dependencies exhibiting a parallel diagonal pattern across the *S. cerevisiae* genome (Methods). Parallel diagonals with strong average dependencies were enriched for duplicated sequences (Extended Fig. 5A). These observations suggested that the genomic language model did not memorize individual repeated elements but modeled the duplication itself. To test this, we generated artificial sequences that include repeats of different lengths and spacing. As shown in Fig. 5B for two repeat lengths of 100, whose repeating nucleotides were spaced as far as 800 bp from each other, the gLM reconstructed the randomly generated repeated sequences correctly with high confidence and exhibited corresponding parallel diagonals. These randomly generated sequences were not part of the natural training sequences, confirming that the gLM has learned the duplication operation. The capacity for identifying duplications strengthened with repeat length, whereby the average dependencies within parallel diagonals longer than 7 nucleotides was significantly higher than background dependencies (Fig. 5B).

Similarly, the gLM captured duplicated sequences in the reverse complement orientation (Fig. 5B, using antiparallel dependencies). Since, as shown in the previous section, antiparallel diagonals reflect stems in RNA structures, we asked whether the gLM captured RNA stems as specific cases of reverse complement duplications. We tested this hypothesis by focusing on all 70 unique tRNA sequences of *S. cerevisiae.* For each tRNA, we generated 100 random sequences of matched length and nucleotide composition in which we inserted at non-overlapping locations an arbitrary sequence and its reverse complement (Methods). As for the same-strand duplications, the gLM captured the reverse-complement relationships with dependencies increasing with repeated element length (Extended Fig. 5C). However, for element lengths matching typical stems in *S. cerevisiae* tRNA arms (2-7 nucleotides), average dependencies on simulated sequences were substantially weaker than average dependencies observed for endogenous tRNA sequences (Extended Fig. 5C). Accordingly, the gLM does not rely only on reverse complementarity alone to link bases in contact and must leverage the broader context of the tRNA sequence. As randomly occurring short reverse complement sequences are pervasive (Extended Fig. 5C) this property reduces false positives in functional contact prediction.
