# Supplementary material for: Nucleotide dependency analysis of genomic language models detects functional elements
Source: Nat Genet. 2025 Oct 10;57(10):2589–602. doi: 10.1038/s41588-025-02347-3 (PMC12513839; doi:10.1038/s41588-025-02347-3)
Supplement: Supplementary file 2 — Reporting Summary [file 41588_2025_2347_MOESM2_ESM.pdf]

nature portfolio

Corresponding author(s): Julien Gagneur

Last updated by author(s): Jul 28, 2025

Reporting Summary

Nature Portfolio wishes to improve the reproducibility of the work that we publish. This form provides structure for consistency and transparency in reporting. For further information on Nature Portfolio policies, see our Editorial Policies and the Editorial Policy Checklist.

Statistics

For all statistical analyses, confirm that the following items are present in the figure legend, table legend, main text, or Methods section.

n/a	Confirmed	
☐	☒	The exact sample size (*n*) for each experimental group/condition, given as a discrete number and unit of measurement
☒	☐	A statement on whether measurements were taken from distinct samples or whether the same sample was measured repeatedly
☐	☒	The statistical test(s) used AND whether they are one- or two-sided<br>*Only common tests should be described solely by name; describe more complex techniques in the Methods section.*
☒	☐	A description of all covariates tested
☒	☐	A description of any assumptions or corrections, such as tests of normality and adjustment for multiple comparisons
☐	☒	A full description of the statistical parameters including central tendency (e.g. means) or other basic estimates (e.g. regression coefficient) AND variation (e.g. standard deviation) or associated estimates of uncertainty (e.g. confidence intervals)
☐	☒	For null hypothesis testing, the test statistic (e.g. *F*, *t*, *r*) with confidence intervals, effect sizes, degrees of freedom and *P* value noted<br>*Give P values as exact values whenever suitable.*
☒	☐	For Bayesian analysis, information on the choice of priors and Markov chain Monte Carlo settings
☒	☐	For hierarchical and complex designs, identification of the appropriate level for tests and full reporting of outcomes
☐	☒	Estimates of effect sizes (e.g. Cohen's *d*, Pearson's *r*), indicating how they were calculated

Our web collection on statistics for biologists contains articles on many of the points above.

Software and code

Policy information about availability of computer code

Data collection	No software was used for data collection.
Data analysis	We used the following packages: pyRanges (v0.0.127), PyTorch (v2.1.0), biopython (v1.81), flash-attn(2.0.4), matplotlib (v3.7.2), numpy (1.24.4), pandas (2.0.3), plotnine (0.12.3), pyfaidx (0.8.1.1), scikit-learn (1.3.0), scipy (1.10.1), seaborn (1.3.0), transformers (4.26.1), cactus (2.9.3), phast, nhmmer (3.1b2), Clustal-Omega (1.2.4), MAFFT (7.525), ViennaRNA (2.6.4).<br><br>For the alignment of DMS-MaPseq reads we used the rf-map module of the RNA Framework and Bowtie2. Count of DMS-induced mutations and coverage and reactivity normalization were performed using the rf-count-genome and rf-norm modules of the RNA Framework.<br><br>Code to reproduce all analysis is available at https://github.com/gagneurlab/dependencies_DNALM or at v410.5281/zenodo.16524884 under MIT license.

For manuscripts utilizing custom algorithms or software that are central to the research but not yet described in published literature, software must be made available to editors and reviewers. We strongly encourage code deposition in a community repository (e.g. GitHub). See the Nature Portfolio guidelines for submitting code & software for further information.

Data

Policy information about availability of data

All manuscripts must include a data availability statement. This statement should provide the following information, where applicable:

- Accession codes, unique identifiers, or web links for publicly available datasets
- A description of any restrictions on data availability
- For clinical datasets or third party data, please ensure that the statement adheres to our policy

As stated in the Data availability section of the manuscript: "Data to reproduce the analysis, together with the 69-Saccharomycetales genome alignment and conservation score, as well as the bacterial and archaeal genomes and the plasmid sequences used for the benchmark against RNAalifold, is provided at https://zenodo.org/doi/10.5281/zenodo.12982536. The SpeciesLM models are available at https://huggingface.co/collections/johahi/specieslms-678a39261cfff01c1fa3ae41. Raw DMS-MaPseq data has been deposited to the Gene Expression Omnibus database (GEO), under accession GSE271937 with code gpizuwsexrqfryd ."

Research involving human participants, their data, or biological material

Policy information about studies with human participants or human data. See also policy information about sex, gender (identity/presentation), and sexual orientation and race, ethnicity and racism.

Reporting on sex and gender	N/A
Reporting on race, ethnicity, or other socially relevant groupings	N/A
Population characteristics	N/A
Recruitment	N/A
Ethics oversight	N/A

Note that full information on the approval of the study protocol must also be provided in the manuscript.

Field-specific reporting

Please select the one below that is the best fit for your research. If you are not sure, read the appropriate sections before making your selection.

☒ Life sciences ☐ Behavioural & social sciences ☐ Ecological, evolutionary & environmental sciences

For a reference copy of the document with all sections, see nature.com/documents/nr-reporting-summary-flat.pdf

Life sciences study design

All studies must disclose on these points even when the disclosure is negative.

Sample size	No sample size calculations were performed as these were predetermined by the datasets we used to analyze the DNA language model.
Data exclusions	We did not exclude any data.
Replication	Not applicable.
Randomization	Not applicable.
Blinding	Not applicable.

Reporting for specific materials, systems and methods

We require information from authors about some types of materials, experimental systems and methods used in many studies. Here, indicate whether each material, system or method listed is relevant to your study. If you are not sure if a list item applies to your research, read the appropriate section before selecting a response.

Materials & experimental systems

n/a	Involved in the study
☒	☐ Antibodies
☒	☐ Eukaryotic cell lines
☒	☐ Palaeontology and archaeology
☐	☒ Animals and other organisms
☒	☐ Clinical data
☒	☐ Dual use research of concern
☒	☐ Plants

Methods

n/a	Involved in the study
☒	☐ ChIP-seq
☒	☐ Flow cytometry
☒	☐ MRI-based neuroimaging

Animals and other research organisms

Policy information about studies involving animals; ARRIVE guidelines recommended for reporting animal research, and Sex and Gender in Research

Laboratory animals	The goal of this experiment is to show experimental supports complementary to all computational evidence and benchmarks of established RNA structures. To this end we performed DMS for E. coli TOP10 cells at 37C. A single biological replicate was produced. This was deemed sufficient as it showed high correlation to previously published DMS-MaPseq data for the same E. coli strain (GSE247244; r = 0.94, over A/C bases with coverage >= 1,000X).
Wild animals	N/A
Reporting on sex	N/A
Field-collected samples	N/A
Ethics oversight	N/A

Note that full information on the approval of the study protocol must also be provided in the manuscript.

Plants

Seed stocks	This study involved no plants.
Novel plant genotypes	N/A
Authentication	N/A
